# Deep Neural Network Recognition of Rivet Joint Defects in Aircraft Products

**DOI:** 10.3390/s22093417

**Published:** 2022-04-29

**Authors:** Oleg Semenovich Amosov, Svetlana Gennadievna Amosova, Ilya Olegovich Iochkov

**Affiliations:** 1 Laboratory of Intellectual Control Systems and Modeling, V.A. Trapeznikov Institute of Control Sciences of Russian Academy of Sciences, 117997 Moscow, Russia; 2 Laboratory of Cyber-Physical Systems, V.A. Trapeznikov Institute of Control Sciences of Russian Academy of Sciences, 117997 Moscow, Russia; amosovasg@yandex.ru; 3 Department of Postgraduate Studies, Komsomolsk-on-Amur State University, 681013 Komsomolsk-on-Amur, Russia; iochkov07@mail.ru

**Keywords:** defect, rivet joint, aircraft equipment, detection, classification, pattern recognition, deep neural network, computer vision

## Abstract

The mathematical statement of the problem of recognizing rivet joint defects in aircraft products is given. A computational method for the recognition of rivet joint defects in aircraft equipment based on video images of aircraft joints has been proposed with the use of neural networks YOLO-V5 for detecting and MobileNet V3 Large for classifying rivet joint states. A novel dataset based on a real physical model of rivet joints has been created for machine learning. The accuracy of the result obtained during modeling was 100% in both binary and multiclass classification.

## 1. Introduction

The rivet joint defectiveness is one of the most sensitive issues for aircraft-building enterprises. This is explained by the growing production volumes and by carrying out many technological operations manually. These defects are detected not only in the workshops of aggregate assembly production but also in the final assembly workshop and, that is worse, at the flight test station, immediately before flight tests. Moreover, the detection of defects should be carried out during the operation of aircraft (AC) as well.

**Figure 1:**
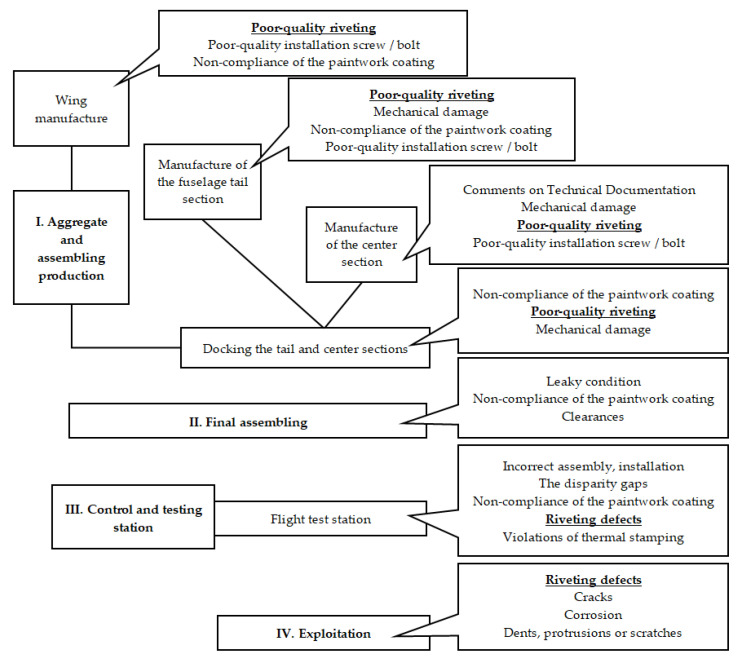
Distribution of inconsistencies identified during quality control at the stages of aircraft items production.

An AC production is made according to the technological documentation (TD) developed on the basis of the requirements of drafts, technical specifications (TS), and directive technologies. The task of the technical control service of the aircraft-building enterprise is to detect defects in the components of its own production at the early stages, in the workshops of aggregate and assembling production, or in the final assembling workshop, until the transfer of the assembled aircraft to the flight test station (Figure 1). All tests of assembling units and aircraft items are often carried out by quality control department specialists manually, by the methods of non-destructive testing. Modern aircraft, both civil and military, have a large number of rivet joints, containing from 10 to 30 thousand rivets. Manual inspection of these rivets is not only laborious but also requires a lot of time and costs. These are difficult tasks for a person, especially when working with small defects when these inconsistencies are barely or not visible to the naked eye.

Therefore, the problem of detecting, monitoring, and diagnosing rivet joint defects requires a current solution.

The rivet joint defects in aircraft items can be of two types: (1) visible, external joint defects; (2) invisible, internal defects. In this article, the object of research is the first type of defect.

In the first case, the defects can be detected with the help of computer vision. There are some GOST standards and production instructions for quality control of holes, sockets, and joints that define such methods and means of monitoring rivet joints as measuring tools, visual inspection, patterns, indicator devices, and probing. Globally, the entire process of rivet joints and holes quality control is based on visual detection of defects using a minimum set of measuring tools: a probe, a ruler, a pattern, and an indicator device. However, there is no ready-made dataset in the public domain for solving the problem of image detecting and recognizing rivet joint defects.

The datasets that are available in the public domain are mainly for the task of detecting and recognizing defects on metal surfaces, such as scratches, dents, recesses, and spots. For example, in 2013, a database of surface defects called NEU-DET was published and created to demonstrate the effectiveness of an approach based on the local binary patterns (LBP) method. The method allows to estimate threshold changes in the neighboring pixels and classify defects of the steel surface according to variations of signs of intra-class changes, illumination, and changes in the shades of gray [1]. The NEU-DET set consists of 1800 images with a definition of 200 × 200 pixels for six different defects of scratches, spots, cracks, and pits on a hot-rolled steel strip. However, the proposed processing method is difficult to apply in real conditions since it requires a complex adjustment limitation for recognizing defects that are sensitive to certain environmental factors, such as lighting and background. When environmental factors change, these thresholds must be carefully adjusted again; otherwise, the algorithm is not applicable in a new environment due to the lack of adaptability and reliability.

In 2020, a new GC10-DET dataset for detecting metal surface defects was proposed to replace NEU-DET [2]. In this paper, two datasets, NEU-DET and GC10-DET, are compared by means of several modern methods: single shot multibox detector (SSD), regions with convolutional neural networks (Faster-RCNN), and you only look once (YOLO-V2), YOLO-V3; traditional methods, such as LBP and histogram of oriented gradients (HOG). The classifiers are used: the k-nearest neighbors algorithm (NNC) and the support vector machine (SVM) methods. As a result of the study, the authors note that there are still serious problems with the GC10-DET dataset with the categories of defects, the number of images, and the scale of data, but the proposed end-to-end defect detection network (EDDN), based on SSD, is resistant to binary detection of defects on metal surfaces.

Recognition of the quality of welds on a metal surface is also a popular direction. There are also many papers concerning this issue and datasets prepared for research works. In [3], a visual system is proposed for measuring the quality of welding and detecting defects. The visual system consists of an image sensor, a laser projector, a focusing lens, and an optical filter. The control system not only measures the size of the weld but also detects weld defects. In addition to the ability to measure the size of the weld and detect defects, the laser quality control system of welds also allows you to reconstruct the three-dimensional surface of the weld.

The effectiveness of computer vision for detecting defects in the welding process is described in [4]. In order to detect faults more thoroughly, it is proposed to estimate 3D scanning models and fluctuations in power consumption during welding, which have template patterns for V-shaped incisions and grease contamination.

Therefore, creating a high-quality dataset for detecting rivet joint defects is a serious and difficult task. Let us consider the works aimed at detecting and recognizing defective rivets on the aircraft skin.

In [5], it is proposed to use a neural network (NN) of direct propagation and a fuzzy system based on perceived shock signals as a riveting control device and detection of riveting gaps at the stages of producing assembly units of aircraft equipment. The fuzzy mechanism plays the role of a signal processor, which is used to improve the important characteristics of shock signals so that the NN can perform an immediate diagnosis of the quality of riveting based on the processed signals.

In [6,7,8], the algorithms for detecting surface defects during aircraft operation are described: cracks and corrosion on the surface of the aircraft skin—direct propagation NN and wavelet filtering on monoscopic and stereoscopic images [6]; dents, protrusions, or scratches on the external surface of the aircraft-3D scanner, segmentation, identification [7]; cracks and scratches of the aircraft body—direct propagation NN, Contourlet Transform (CT) and Discrete Cosine Transform (DCT) [8].

In order to check aging aircraft, the method of non-destructive testing with Magneto-Optical Imaging (MOI) is used, it is used to detect surface and subsurface cracks and corrosion of the aircraft skin [9]. The main advantage of magneto-optical imaging technology is a quick check over a large area. The interpretation of images obtained by the magnetic-optical method is simple but still depends on the human factor and fatigue, which leads to a spread in the level of confidence in the probability of detection (POD). In this paper, the automated control is proposed to detect rivets and defects, classify defects and decide on acceptance or rejection based on magneto-optical scanning, conversion to a black-and-white image, and the use of morphological image processing to find the center and radius of the rivet object on the surface. If the diameter of the found object is close to the size of the rivet and the image contains several non-zero pixels in the center of the rivet, it means that this is a “good” rivet, while the abnormal rivet has an additional “drop” that is located outside the detected circle. Therefore, to determine the “width” of the additional area outside the rivet in the image, two asymmetry functions are used that determines the distance from the edge to the center of the rivet. In [10], the authors refined the detection of the area outside the rivet circle using the Hough Transform.

Hidden rivets are difficult to be distinguished on a metal surface in video images due to glare and reflection. Therefore, the authors of [11] developed a three-dimensional sensor to solve the problem of determining the rivet contour. The 3D sensor consists of two rigidly directed video cameras and a projector in the center, creating a dense three-dimensional image, which allows you to determine the size and exact diameter of the rivet. The Fringe Projection Profilometry (FPP) algorithm is used for segmentation, and the points of the rivet edges are further refined by the High Dynamic Range (HDR) algorithm. This work is aimed at detecting the edge of the rivet on the metal surface, but there is no solution for determining the defects of the rivet, although the results of the work are impressive.

In the development of research in the field of 3D scenes, an automated quality check of countersunk rivets in the aircraft structure is proposed in [12]. A mobile 3D scanning system has been developed for automation, and a deep convolutional neural network Rivet Region Classification Network (RRCNet), has been created for the detection and classification of rivets. This article is aimed at solving the problem of detecting hidden rivets on the surface of the aircraft skin since they are poorly distinguished on the surface; therefore, due to 3D scanning, a side view of the rivet is obtained. Although this paper emphasizes the need for 3D scanning to collect information about the shape of the aircraft skin and control the rivets, the process of detecting and recognizing the rivet is carried out on 2D projection maps with images of a point cloud. The proposed method is good for comprehensive coverage of the entire viewing angle, which eliminates the omission or false detection of hidden rivets.

Similar work was started by us in 2019 [13,14]. Our presentations at the conferences [13,14] are only a conceptual, preliminary analysis set out on a few pages. This manuscript provides theoretical, mathematical, physical, and practical research that significantly goes beyond the presentations at the conferences [13,14]. The novelty in the obtained results of deep neural network recognition and multiclass classification of defects using a new dataset on rivet connections based on real defects is presented.

## 2. The Statement of the Task of Detecting and Classifying the Defects

The tested product is considered, the condition of which can be classified as serviceable or defective. We introduce a finite set $B=\{\beta_{1},\ldots ,\beta_{\mu },\ldots ,\beta_{\eta }\}$, containing the η state classes of the controlled item. Its elements are a class of states that characterize the serviceable state of the product and several classes of functional states that characterize the defective state.

It is necessary to detect the images of objects according to the incoming video stream by highlighting key features and assigning them to one of the classes. In order to implement image recognition, it is necessary to develop a computational method based on both traditional and machine learning methods, including modern deep neural networks, and conduct a full-scale experiment with a video stream coming from surveillance cameras of the aircraft equipment. 

Assume that we have:

(1) a multitude of images ω∈Ω, each of which is represented as a 2*D* set of the images $I^{j}$, $j=\bar{1,M}$ and is characterized by the features $x_{i}$, $i=\bar{1.m}$, the set of which for the image ω is represented by the vector description $x=\Phi \left(\omega \right)={\left(x_{1}\left(\omega \right),x_{2}\left(\omega \right),\ldots ,x_{m}\left(\omega \right)\right)}^{T}$; 

(2) a multitude of product condition classes $B=\{\beta_{1},\ldots ,\beta_{\mu },\ldots ,\beta_{\eta }\}$, η—the number of classes. 

A priori information is represented with the training set $D=\left\{\left(I^{j},\beta^{j}\right)\right\},j=\bar{1,L}$, given by the table, in which each line j contains a 2*D* image $I^{j}$ and the class label $\beta_{\mu }$, $\mu =\bar{1,\eta }$. It should be noted that the training set characterizes the unknown display *F:Ω→B.

It is necessary to solve the problem of image recognition according to available frames $I_{t}$ of the continuous video stream $V=\left(I_{1},..,I_{t},..,I_{\tau }\right)$ and a priory information given by the training set $D=\left\{\left(I^{j},\beta^{j}\right)\right\},j=\bar{1,L}$ for the deep machine learning with a teacher: to detect the images ω as the features estimation $\tilde{x}$ with the help of the display $F_{1}:I_{t}\to \tilde{x}$ and to classify them by using the display $F_{2}:\tilde{x}\to \beta_{\mu }$, $\mu =\bar{1,\eta }$ in accordance with the given criterion $J\left(\tilde{x}\right)$ minimizing the error probability [15,16].

Thus, it is necessary to find the display $F_{3}:I_{t}\to \beta_{\mu }$, $\mu =\bar{1,\eta }$, by which $F_{3}$ is the set of functions and algorithms $f_{i}$, $i=\bar{1,N_{f}}$. 

It should be noted that the simplest task of binary classification is the classification when assigning the presented image to one of two classes {“absence of a defect”, “presence of a defect”}, $\beta_{\mu }\in B=\{0;\;1\}$. However, in the tasks of recognizing product defects, the desired solution is precisely a multiclass classification with an understanding of the nature of the defects. This is necessary for the application of the methods for their elimination and subsequent prevention during the process of production.

## 3. Computational Method Based on the Deep Neural Networks of Machine Learning

The detection of rivet defects can be carried out using various methods, such as the Circle Hough Transform (CHT), morphological image processing, and 2-D dimensional convolution [9,10,11,12].

Recently, one of the most noticeable trends has been to use deep neural networks in solving image recognition problems [15,16,17]. They are assigned the main role in the proposed method.

In order to detect and classify objects and defects, it is offered to use the computational method of image recognition $F_{3}:I_{t}\to \beta_{\mu }$, $t=\bar{1,\tau }$, $\mu =\bar{1,\eta }$ with its implementation based on deep neural networks. 

The computational method consists of the following stages:

(1) The rivet joint is localized on the frame $I_{t}$ of the continuous video stream $V=\left(I_{1},..,I_{t},..,I_{\tau }\right)$. This sub-task is offered to be solved by the pre-trained architecture of the deep NN YOLO for object detection [18]. The result of the work is the Region of Interest (ROI) containing the image of the object. This improves the performance and simplifies the algorithms for the subsequent defects recognition.

(2) For multiclass classification of the rivets, state informative features are distinguished using deep machine learning, for example, using a deep convolutional neural network trained on the datasets $D^{ML}$ of defective and normal rivets prepared by us.

(3) Assignment of the feature vector to one of the classes. The classification criterion is defined as $J(\tilde{x})=\max p(\tilde{x})$. If $J(\tilde{x})\geq \varepsilon$, where ε—the given limitation, then $\beta_{\mu }=\underset{\mu \in 1..\eta }{\arg\max}(p(\tilde{x}))$, otherwise the classification is considered to be incorrect.

## 4. Automated Workplace of the Control Operator. Creating a Dataset

**Figure 2:**
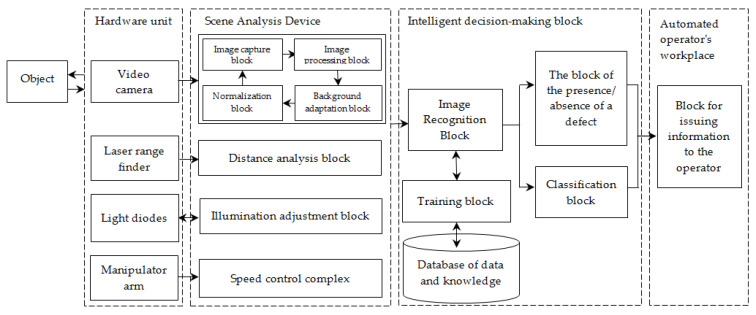
The system for recognizing rivet joint defects.

In order to control the quality of rivet joints, it is proposed to use an automated workplace for the control operator in the aggregate assembly production workshop of an aircraft-building enterprise (Figure 2).

**Figure 3:**
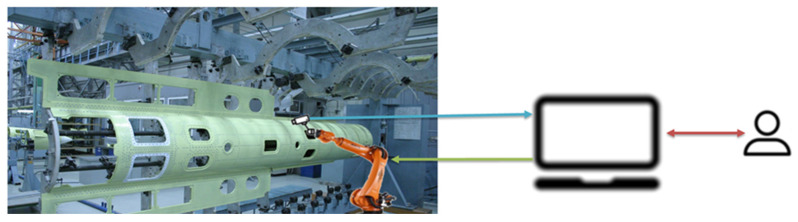
The sketch design of the automated workplace for the control operator of the aggregate and assembly production workshop.

The sketch design of the automated workplace for the control operator of the aggregate and assembly production workshop consists of a manipulator arm that moves along the surface specified in the design documentation; video cameras record the quality of holes, sockets, rivet connections; the recognition system based on the computational method compares them with the existing database of defects (Figure 3). When a defect is detected, this place on the surface is fixed, and information is transmitted to the control system operator (the type of defect, recommendations for elimination).

The fuel tank panel is located in the slipway, and the manipulator arm moves along a preplanned path in accordance with the design documentation. There are video cameras at different angles, laser rangefinders, and LEDs on the manipulator arm. Laser rangefinders are necessary for monitoring the shooting distance. The accuracy of defect detection is affected by many external factors: the direction of lighting, blinking, reflection, darkening, and the angle. Therefore, the LEDs create directional lighting to eliminate external factors on the examined surface. Video cameras also serve as primary sources of information. The video stream of controlled rivets received from them enters the information pre-processing unit, then the system detection and classification unit, and then the processed and analyzed information is sent to the operator’s monitor with recommendations for eliminating defects in order for the operator to make the right decision.

**Figure 4:**
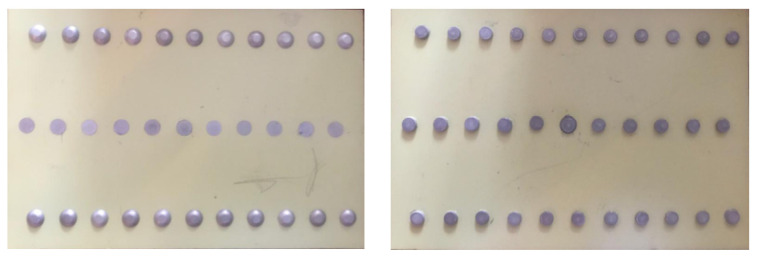
An example of plates with rivets.

In order to implement the computational method, model it, and check its accuracy, it is necessary to form a set of defect classes. For this purpose, duralumin plates were prepared (Figure 4), consisting of two halves riveted together with various types of rivets, both with countersunk and barrel-shaped (flat-rounded) heads.

**Figure 5:**
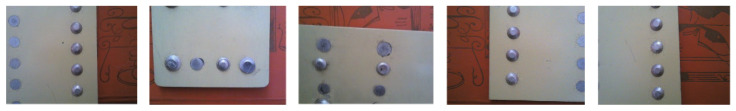
Sample images for training the object detector and for testing the classifier.

The dataset for detecting rivets includes 375 images of duralumin plate fragments with barrel-shaped and countersunk rivets (Figure 5). Images of plate fragments contain images of normal and defective rivets, and full images of rivets and their individual parts.

A total of 345 images were used to train the object detector based on the YOLO-V5 deep NN [19], and 15 images were used for testing and validation. These images were annotated in Pitch YOLO-V5 format.

The following pre-processing was applied to each image: auto-orientation of pixel data (with EXIF-orientation stripping). The following augmentation was applied to create three versions of each source image: (1) 50% probability of horizontal flip; (2) randomly crop between 0 and 20 percent of the image. The following transformations were applied to the bounding boxes of each image: salt and pepper noise was applied to five percent of pixels.

The marking of images was carried out using the Computer Vision Annotation Tool (CVAT, [20]) service and was performed manually, using the designation of objects in each image. A metafile with coordinates was formed in the following format: Label_ID X_CENTER Y_CENTER WIDTH HEIGHT. For each <image_name>.jpg image, an <image_name>.txt file with markup was created.

**Figure 6:**
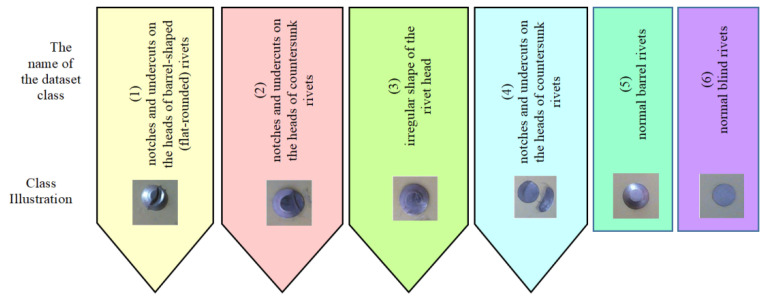
The structure of rivet joints dataset.

After that, photos of non-defective and defective rivets of various types were taken to prepare the classifier dataset. The collected rivet joints dataset contains two classes of non-defective and four classes of defective rivets (Figure 6), namely: notches and cuts on the barrel-shaped (flat-rounded) rivet heads (1); notches and cuts on the countersunk rivet heads (2); the irregular shape of the embedded head of the rivet (3); cuts and risks on the surface of the part (4); normal barrel-shaped rivets (5); normal countersunk rivets (6). 

The dataset for the rivet classifier consists of 200 images for each class. A total of six classes (1200 images) were prepared to test the classification (768 images for training, 192 images for validation, and 240 images for test). The described datasets for detecting and classifying rivets can be found on GitHub [21].

## 5. The Realization of the Computational Method

Let us consider the main stage features of the computational method.

### 5.1. Localization and Detection of a Rivet Joint in an Image Using YOLO-V5

When choosing YOLO-V5, we were guided by studies conducted in [22]. The results of the study are presented in Table 1.

Table 1 shows the results of applying the YOLO algorithms to a sample image, demonstrating the accuracy and completeness of these algorithms. YOLOv3 has high accuracy, but its recall is low, which shows that the model needs to be improved. We see that the models in YOLOv4 and YOLOv5 have a balanced accuracy and recall resulting in a high F1 score [22].

Let us reflect on the differences between the YOLOv3, YOLOv4, and YOLOv5 architectures. The YOLOv3 architecture uses the backbone Darknet53. The YOLOv4 architecture uses CSPdarknet53, i.e., CSNet in Darknet, and the YOLOv5 architecture uses the Focus structure with CSPDarknet53. The advantage of using the Focus layer is to reduce the required CUDA memory, reduce the number of layers and improve speed and accuracy [19].

As an algorithm for detecting rivets, it is proposed to use the YOLO-V5 deep neural network architecture. To train YOLO-V5, we use a dataset prepared by us with images of fragments of plates with rivet connections (Figure 5). The detection result is the marking of the image on the area of the found rivets, i.e., we obtain an array containing their coordinates and the probabilities of finding the object. 

Table 2 shows the values of the rivet detection metrics with the best weights at the end of the YOLO-V5 NN training. Here you should pay attention to the values of the precision and recall metrics and two variants of the mAP metric (mean Average Precision for object detection).

The average training time for the YOLO-V5 deep NN is about 16 min on the i5 7400 Geforce 1080Ti PC configuration, Ubuntu Linux operating system. The time to check the quality of detection (validation) is about 99 s.

**Figure 7:**
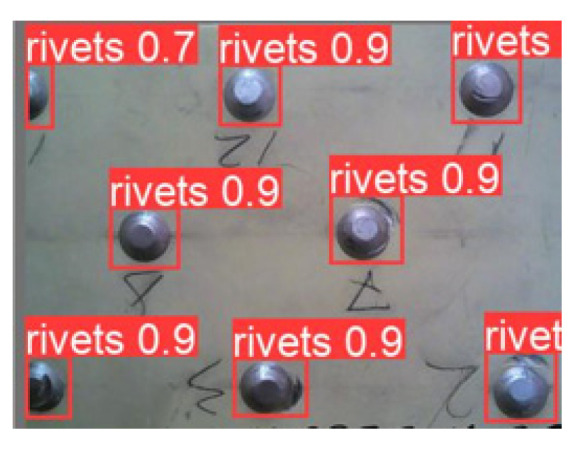
The result of rivets detection with confidence values on the validation set.

To check the accuracy of the work and the prediction of the proposed model, we use the reliability indicator—the Confidence metric, which reflects the “confidence” of the model that an object has been detected or not. The validation was carried out on an additional dataset, which is 20% in relation to the prepared dataset for object detection. The result of the model’s work on the validation set is shown in Figure 7.

**Figure 8:**
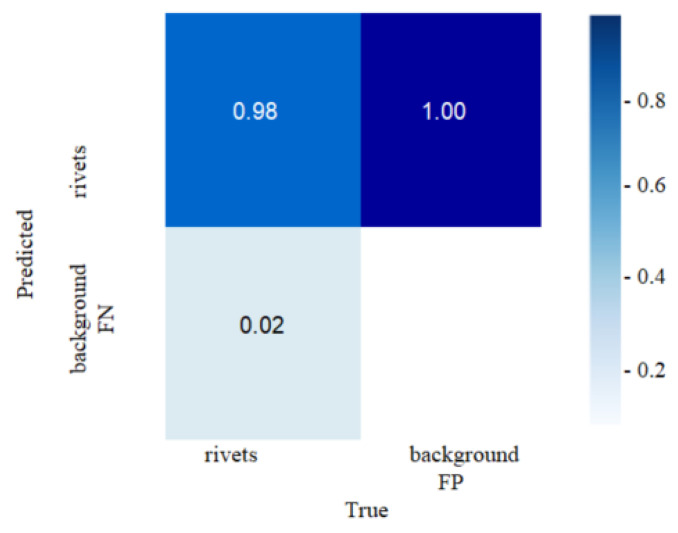
Confusion matrix.

Figure 7 shows that there are no gaps and recognition errors. As a result of the work of the YOLO-V5 deep NN, we obtain images with the designations of the detected objects and confidence values. In order to reflect the accuracy and completeness, the confusion matrix is calculated (Figure 8).

The confusion matrix shows that when rivets are detected on a fragment of the plate, an error of 2% appears due to the confusion of the rivet with the metal surface (with the background of the plate). In this case, the test was carried out on 15 images, including 105 rivets of various types of the complete type and their individual parts.

As can be seen from the matrix in Figure 8, most of the rivets are detected correctly.

The F1-score metric, which combines information on the accuracy and completeness of the rivets detection model and is calculated by the formula:

$$F1=2\frac{\mathrm{Precision}\times \mathrm{Recall}}{\mathrm{Precision}+\mathrm{Recall}}.$$

As a result, the detection of rivets on the surface of panels using YOLO-V5 is 98%. However, for the classification of defects into classes, the use of this deep NN is not advisable since it gives a high level of errors. Therefore, to classify defects, it is proposed to use another convolutional deep neural network trained on higher resolution images, which makes it possible to determine the object class more reliably.

### 5.2. The Peculiarities of Using CNN for Detecting Rivet Joint Defects

In the considered papers [5,6,7,8,9], feedforward neural networks are used to detect defects in rivets, rivet holes, and surfaces. Since we have analyzed these papers, it is possible to point out the problems of using such NN:

- a large amount of images of subassemblies of various designs is required for training;
- there is no possibility to change the scale of the input images, their angle, and other affine-geometric transformations;
- the images must be of high quality to display all small defects, and this leads to an increase in the dimension and processing time;
- the use of several neural networks for different input data leads to an increase in computational complexity.

Therefore, deep convolutional neural networks (DNN) were chosen to solve the problem of detecting defects since they provide work with huge data arrays, partial resistance to changes in scale, displacements, rotations, angle changes, and other distortions. 

**Figure 9:**
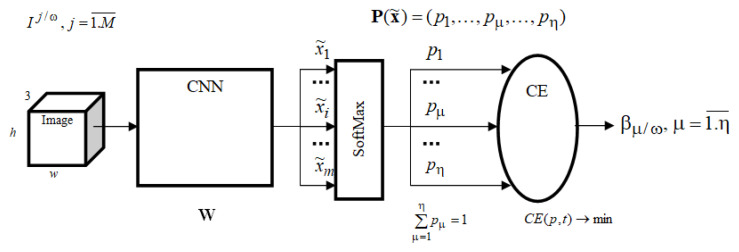
General structure CNN.

The general structure of the defect recognition system for multiclass identification based on deep machine learning using NN is shown in Figure 9: The general structure of the DNN.

The ideology of the convolutional neural network architecture is based on some features of the human visual cortex. Namely, it is based on a similar apparatus of simple and complex cells, i.e., filters (nuclei) that highlight straight lines, arcs, borders, contrast, and activation functions that enhance the effect of filters on the image.

The idea of convolutional neural networks is to alternate convolution layers and subsampling layers (or pooling Layers, subsampling layers).

When training the NN with an output SoftMax layer, a loss function in the form of cross-entropy is used for multiclass classification:

$$CE(w)=\sum_{n=1}^{N}CE_{n}(w)=-\sum_{n=1}^{N}\sum_{\mu =1}^{\eta }t_{n,\mu }\log(SM_{\mu }({\tilde{x}}_{n},w)),$$

where $t_{n,\mu }$—the target value for the class μ, the class label corresponding to the *n* example (it is equal to zero for all the classes except the class μ, for which this value is equal to 1); $p_{\mu }=SM_{\mu }({\tilde{x}}_{n},w)$—the probability of this class is equal to the value of the SoftMax function from the outputs of the neural network; η—the number of classes; *N*—the size of the training set (the number of examples); ${\tilde{x}}_{n}$—*m*-vector of the features formed by the network at the SoftMax layer input, $n=\bar{1,N}$; w—the vector of configurable weight coefficients of the neural network.

SoftMax activation function:

$$SM_{i}(\tilde{x})=\frac{e^{{\tilde{x}}_{i}}}{\sum_{j=1}^{n}e^{{\tilde{x}}_{j}}},\;0<SM_{i}<1,\;\sum_{i=1}^{n}SM_{i}(\tilde{x})=1.$$

To estimate the classifier, a generally accepted metric is also used—general accuracy $M^{Ac}$ (Accuracy). The value of the metric $M^{Ac}$ is calculated as:

$$M^{Ac}=\frac{TP+TN}{TP+TN+FP+FN},$$

where *TP*—True Positive rate, *TN*—True Negative rate, *FP*—False Positive rate, *FN*—False Negative rate [23].

As a neural network classifier, we have proposed a simple deep NN architecture built by various combinations of convolution layers. The network begins with an input sequence layer, followed by three blocks in a row, consisting of a convolutional layer with a RELU activation function and a subsampling layer with various empirically selected parameters. In order to predict class labels, the network ends with a fully connected layer, a SoftMax layer, and a classification output layer. The NN architecture was trained and tested on i5 7400 Geforce 1080Ti PC configuration, Ubuntu Linux operating system, using the created dataset for classification.

The given example of the convolutional NN shows that if we use not complex convolutional neural networks, then with two class recognition, their using gives 100% defect detection, and with multiclass recognition, the accuracy is 97% for six classes. Errors occur when a defect is assigned to a neighboring, close defect class and not to a serviceable state. Therefore, it is proposed to use a new generation of highly accurate and efficient neural network models for multiclass classification.

Of the latest achievements in the field of deep learning, it is worth noting the MobileNet NN architecture, which has low requirements for computing resources and has not been previously used for the problem of recognizing defects in rivet joints.

The MobileNet V3 Large deep NN [24] was retrained by us using our own marked dataset and modified for the task being solved.

**Figure 10:**
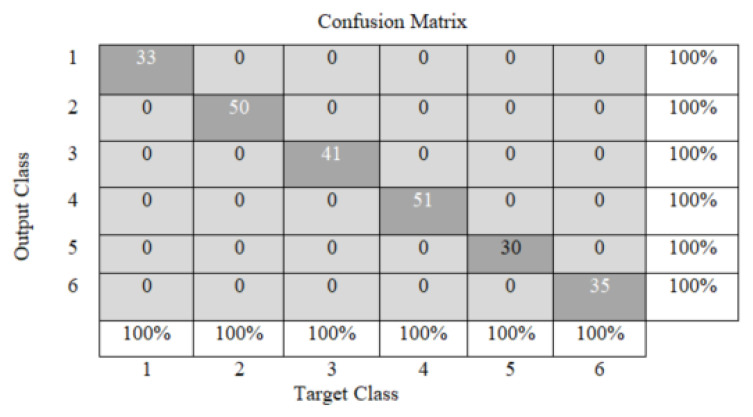
Confusion matrix in testing MobileNet V3 Large.

To solve the problem of classifying defects in rivet joints, we modified the basic architecture of MobileNet V3 Large as follows (Figure 10, Table 3):

- in the output layer (Dense) of the last block, the output vector was changed to a size of 1 × 6 to bring it to the problem of assignment to one of the six classes;
- the modified NN has been retrained to classify defects in riveted joints.

We also made a comparison with the architecture of CNN MobileNet V3 Small. The results are shown in Table 4. From them, it can be concluded that the used CNN MobileNet V3 Small is not so effective in classifying defects. However, it is worth noting that the training time of CNN MobileNet V3 Small is several times less than CNN MobileNet V3 Large.

**Figure 11:**
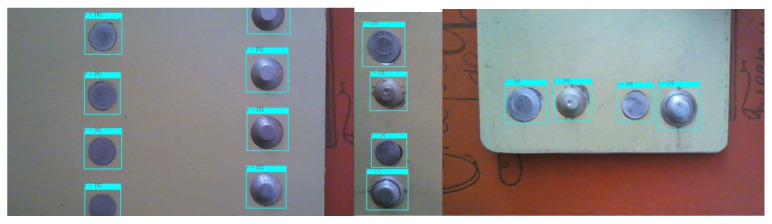
Test result.

In order to test the software package for detecting and classifying defects in rivet joints, images of duralumin plates with riveted joints were presented (Figure 11). The operation of the detector and the classifier is displayed on the presented image as a highlighted rectangle around the rivet area with the class number indicated. The accuracy of the six-class classification on the test dataset was 100%, but the detection of rivets on the surface of panels using YOLO-V5 is 98%; therefore, the accuracy of defect classification does not exceed 98%.

## 6. Conclusions

The mathematical statement of the problem of recognizing and classifying rivet joint defects in aircraft products is given. 

A computational method for the recognition of rivet joint defects in aircraft equipment based on video images of aircraft joints has been proposed with the use of the YOLO-V5 NN for detecting and MobileNet V3 Large NN for classifying rivet joint states.

A novel dataset based on a real physical model of rivet joints has been created for machine learning.

The accuracy of the result obtained during modeling was 100% in the case of binary classification and 100% with the six-class recognition of the rivet joint defects with their 98% detection on the surface of the boards.

The use of computer vision together with artificial intelligence technologies will also allow for the detection of abnormal situations in the production process in proper time, recognizing objects and situations in a continuous video stream, and tracking violations occurring during the production and on the territory of the organization.

## Figures and Tables

**Table 1:** Results of comparing YOLOv3, YOLOv4 and YOLOv5.

Measure	YOLOv3	YOLOv4	YOLOv5
Precision	0.73	0.69	0.707
Recall	0.41	0.57	0.611
F1-score	0.53	0.63	0.655
mAP	0.46	0.607	0.633
PC Speed	63.7	59	58.82
Jetson Speed	7.5	6.8	5

**Table 2:** Metrics of the learning process.

Parameters	Precision	Recall	F1-Score	mAPval@0.5	mAPval@0.5:0.95
Yolov5 (78 epochs, 288 labels, val. labels 99)	0.98	0.99	0.985	0.993	0.689
Yolov5 (79 epochs, 296 labels)	0.98	1	0.989	0.993	0.721
Yolov5 (80 epochs, 244 labels)	0.98	0.99	0.985	0.993	0.686
Yolov5 (100 epochs, 345 labels)	0.98	0.98	0.978	0.993	0.708

Note: labels—the number of rivets on the images of plate fragments that participated in the training of the epoch; val. labels—the number of rivets on the images that participated in the validation.

**Table 3:** Classification Report MobileNet V3 Large.

Class	Precision	Recall	F1-Score	Support
1	1.00	1.00	1.00	33
2	1.00	1.00	1.00	50
3	1.00	1.00	1.00	41
4	1.00	1.00	1.00	51
5	1.00	1.00	1.00	30
6	1.00	1.00	1.00	35

**Table 4:** Classification Report MobileNet V3 Small.

Class	Precision	Recall	F1-Score	Support
1	1.00	1.00	1.00	33
2	1.00	1.00	1.00	50
3	1.00	1.00	1.00	41
4	1.00	0.98	0.99	51
5	1.00	1.00	1.00	30
6	0.97	1.00	0.99	35

## Data Availability

Author’s datasets. Available online: https://github.com/Iochkov (accessed on 6 April 2022).
